# Supplementary material for: Structure of human MUTYH and functional profiling of cancer-associated variants reveal an allosteric network between its [4Fe-4S] cluster cofactor and active site required for DNA repair
Source: Nat Commun. 2025 Apr 16;16:3596. doi: 10.1038/s41467-025-58361-w (PMC12000561; doi:10.1038/s41467-025-58361-w)
Supplement: Supplementary file 1 — Supplementary Information [file 41467_2025_58361_MOESM1_ESM.pdf]

SUPPLEMENTARY INFORMATION for:

Structure of human MUTYH and functional profiling of cancer-associated variants reveal an allosteric network between its [4Fe-4S] cluster cofactor and active site required for DNA repair

Carlos H. Trasviña-Arenas[1#], Upeksha C. Dissanayake[2], Nikole Tamayo[1,3], Mohammad Hashemian[1,3], Wen-Jen Lin[1,3], Merve Demir[1,3], Nallely Hoyos-Gonzalez[1], Andrew J. Fisher[1,3,4], G. Andrés Cisneros[2,5*], Martin P. Horvath[6*] & Sheila S. David[1, 3*]

[1]Department of Chemistry, University of California, Davis, California, 95616

[2]Department of Chemistry and Biochemistry, University of Texas at Dallas, Richardson, TX 75801, USA

[3]Chemistry and Chemical Biology Graduate Program, University of California, Davis, CA 95616

[4]Department of Molecular and Cellular Biology, University of California, Davis, California 95616, USA

[5]Department of Physics, University of Texas at Dallas, Richardson, TX 75801, USA

[6]School of Biological Sciences, University of Utah, 257 S 1400 E, Salt Lake City, Utah, 84112

*Corresponding authors: ssdavid@ucdavis.edu, martin.horvath@utah.edu, andres@utdallas.edu.

[#]Current address: Research Center on Aging, Center for Research and Advanced Studies (CINVESTAV), Mexico City 14330, Mexico.

TABLE OF CONTENTS

Supplementary Tables:

Supplementary Table 1. Mutation suppression activity of cancer-associated mutants within the [4Fe-4S] cluster of MUTYH.

Supplementary Table 2. Adenine Glycosylase Activity, Substrate and Product Analog Affinity, Metal analysis of cancer-associated mutants within the [4Fe-4S] cluster of MUTYH.

Supplementary Table 3. Adenine Glycosylase Actvity of WT GsMutY and R149Q GsMutY.

Supplementary Table 4. Data collection and model refinement statistics for MUTYH-TSAC and R149Q Gs MutY structures.

Supplementary Table 5. Reduction of restraints throughout equilibration during molecular dynamic simulations.

Supplementary Table 6. Molecular dynamic simulations system setup

Supplementary table 7. Oligonucleotides used in this study

Supplementary Figures:

Supplementary Figure 1: Multiple sequence alignment of MutY/MUTYH amino acid sequences.

Supplementary Figure 2: Design and details of MUTYH constructs used in this work.

Supplementary Figure 3: Structural comparison of mMutyh/MUTYH.

Supplementary Figure 4. X-ray absorption near-edge spectra of the iron of the [4Fe–4S] cluster and the Zn of MUTYH crystal.

Supplementary Figure 5.- Conservation of structural interplay between the [4Fe-4S] cluster and the active site in Helix-hairpin-Helix DNA glycosylases.

Supplementary Figure 6. SDS-PAGE showing the MBP-MUTYH variants used in this work.

Supplementary Figure 7.- Qualitative glycosylase characterization of MBP-MUTYH cancer-associated mutants.

Supplementary Figure 8. Qualitative binding assays.

Supplementary Figure 9.- Structural analysis of the alternative conformations of the [4Fe-4S] cluster within the R149Q GsMutY-THF:OG structures.

Supplementary Figure 10. Molecular dynamic simulations of WT MUTYH, N238S and R241Q cancer-associated mutants in human structure.

Supplementary Figure 11. Energy decomposition analysis of the nonbonded interactions of the MUTYH human structure with respect to the [4Fe-4S] cluster.

Supplementary Figure 12. Molecular dynamic simulations of WT, N209S and R212Q cancer-associated mutants in mMutyh mouse structure.

Supplementary Figure 13. Additional results of the molecular dynamic simulations of WT, N238S and R241Q cancer-associated mutants in mouse Mutyh structure.

Zip file contains the Multiple Sequence Alignment used for MutY/MUTYH coevolutionary analysis.

Supplementary Methods:

Synthesis of 1N phosphoramidite

Supplementary Tables

**Supplementary Table 1**: Mutation suppression activity[a] of cancer-associated mutants within the [4Fe-4S] cluster of MBP-MUTYH.		
MUTYH	Mutation frequency ($f \times 10^8$)	Relative change to WT
Empty vector (pMAL)	5.8(4.68-6.9)	52
WT	0.1(0.08-0.1)	1
W103C	5.1(3.14-7.2)	46
W103R	5.4(3.36-7.4)	48
N238S	1.3(0.34-2.2)	12
R241Q	2.0(1.21-2.8)	18
R241W	4.2(2.65-5.8)	38
R245H	2.6(1.74-3.4)	23
R245C	3.7(2.99-4.4)	33
V246F	0.6(0.41-0.7)	5
C290W	6.1(2.43-9.8)	55
C306W	5.1(3.54-6.6)	46
R309C	1.1(0.4-1.9)	10
[a]Mutation frequency values are shown with 95% confidence limit based on the median value. Source data are provided as a Source Data file.		

**Supplementary Table 2**. Adenine Glycosylase Activity[a], Substrate and Product Analog Affinity[a] ,metal analysis[b] of cancer-associated mutants within the [4Fe-4S] cluster of MBP-MUTYH.							
MUTYH	[4Fe-4S] load	Zn load	k_2 (min^{-1}) A:OG	k_3 (min^{-1}) A:OG	Active MUTYH	k_D (nM) fA:OG	k_D (pM) THF:OG
WT (MBP free)	5.1±0.1	1.2±0.1	1.3±0.1	0.01±0.001	56-35%	ND	ND
WT	4.4±0.1	0.2 ±0.1	1.2±0.1	0.02±0.01	20 %	32±4	<10
W103C	0.0	0.0	ND	ND	ND	ND	ND
W103R	0.0	0.0	ND	ND	ND	ND	ND
N238S	4.1±0.2	0.3±0.1	ND	ND	ND	124±10	46±10
R241Q	4.4±0.1	0.1±0.02	ND	ND	ND	629±80	467±70
R241W	0.0	0.0	ND	ND	ND	ND	ND
R245H	0.0	0.0	ND	ND	ND	ND	ND
R245C	0.0	0.0	ND	ND	ND	ND	ND
V246F	3.9±0.5	0.2±0.1	1.2±0.1	0.01±0.004	27%	50±11	<10
C290W	0.0	0.0	ND	ND	ND	ND	ND
C306W	0.0	0.0	ND	ND	ND	ND	ND
R309C	4.9±0.3	0.2±0.1	1.0±0.1	0.01±0.01	9%	246 ±32	<10

[a]kinetic parameters and dissociation constants were measured as described in the methods. [b]Metal analysis was conducted using Inductively Coupled Plasma-Mass Spectrometry (ICP-MS). Values equal to 0 means that the protein sample had Zn and Fe content equal to the blank. ND; not determined. **Source data are provided as a Source Data file.**

**Supplementary Table 3:** Adenine Glycosylase Activity of WT GsMutY and R149Q GsMutY.				
Enzyme	Active fraction (%)	k_2 (min^{-1}) OG:A	k_2 (min^{-1}) OG:P	k_3 (min^{-1}) OG:A
WT Gs MutY	39±1[b]	54±4[b]	1.1±0.1[c]	0.04±0.01[d]
R149Q Gs MutY	47±5	1.1±0.6	0.2±0.1	0.007±0.001

[a]The glycosylase assays conducted to measure k_2 values were done at 60 °C with 165 nM enzyme, 20 nM radiolabeled DNA, and 30 mM NaCl. The glycosylase assays conducted to measure k_3 values were done at 60 °C. [b,c,d] Previously reported[4-6].

Supplementary Table 4.- Data collection and refinement statistics for MUTYH-TSAC and R149Q GsMutY structures.

Data collection statistics		
**PDB ID**	**8FAY**	**9BS2**
Protein	MUTYH-TSAC	R149Q GsMutY
DNA	OG:N1	OG:THF
Wavelength (Å)	0.97918	0.97741
*Resolution range (Å) **	60.3–1.91	70.3–1.51
Highest resolution shell (Å)	(2.02–1.91)	(1.55–1.51)
Space group	$P2_12_12_1$	$P2_12_12_1$
Unit cell a (Å)	87.686	37.500
b (Å)	116.422	86.000
c (Å)	118.378	140.640
*Total reflections***	484,096 (66,132)	66,1741 (31,326)
Unique reflections	177,017 (27,556)	136,096 (9,284)
Multiplicity	2.7 (2.4)	4.8 (3.4)
Completeness (%)	97.6 (94.1)	98.7 (90.9)
Mean I/sigma(I)	7.53 (0.56)	12.2 (0.51)
Wilson B-factor ($Å^2$)	44.2	33.6
R-merge (%)	8.5 (177.9)	5.4 (221.1)
R-rim (%)†	10.4 (221.3)	6.1 (256.7)
CC1/2 (%)	99.8 (20.9)	99.9 (12.4)
* Statistics for the highest-resolution shell are shown in parentheses.<br>** Friedel mates treated as different reflections<br>† Redundancy independent measure of R[9]		
**Refinement statistics**		
**PDB ID**	**8FAY**	**9BS2**
Protein	MUTYH-TSAC	R149Q GsMutY
DNA	OG:N1	OG:THF
*Resolution range (Å) **	60.3–1.91	70.3–1.51
Highest resolution shell (Å)	(1.98–1.91)	(1.56–1.51)
*Reflections***	174,855 (14,498)	132,137 (12,247)
R-work	0.180 (0.395)	0.207 (0.424)
R-free	0.208 (0.399)	0.230 (0.428)
Protein:DNA per asymmetric unit	2	1
TLS Groups	N.A.	3
Atoms, non-hydrogen	7492	3477
Protein	6102	2783
DNA	844	422
OG nucleotide	46	23
Active site nucleotide	22	11
SF4	16	16
Sulfate	65	N.A.
Calcium	N.A.	3
Solvent	465	249
RMSD bonds (Å)	0.012	0.012
RMSD angles (°)	1.138	1.179
Ramachandran favored (%)	97.7	97.4
allowed (%)	2.3	2.3
outliers (%)	0.0	0.3
Rotamer outliers (%)	0.5	0.7
MolProbity Score	1.02	1.32
Clashscore	2.08	4.24
Average B non-H ($Å^2$)	46.9	41.8
Protein ($Å^2$)	44.9	43.2
DNA ($Å^2$)	58.1	35.2
OG nucleotide ($Å^2$)	34.0	20.9
Active site nucleotide ($Å^2$)	45.6	23.1
SF4 ($Å^2$)	33.7	23.8
Sulfate ($Å^2$)	68.6	N.A.
Calcium	N.A.	38.2
Solvent ($Å^2$)	49.6	38.1
** Friedel mates treated as different reflections		

Supplementary Table 5: Reduction of restraints throughout equilibration during molecular dynamic simulations.

Restraints (kcal mol^{-1} Å^{2})	Time (ns)
500	0.1
400	0.1
300	0.1
200	0.1
100	0.1
50	0.1
25	0.1
10	0.1
5	0.1
2	0.1
1	0.1
0.5	0.1
0	0.5

Supplementary Table 6: Molecular dynamic simulations system setup

**System**	**Simulation box dimensions (Å x Å x Å)**	**Total number of atoms (without water)**	**Total number of atoms (with water)**	**Salt concentration**
WT human	114.64 x 98.44 x 76.67	7221	71448	50mM KCl
N238S	114.64 x 98.44 x 76.67	7218	71445	50mM KCl
R241Q	114.64 x 98.44 x 76.67	7213	72477	50mM KCl
WT mouse	111.69 x 87.37 x 78.92	7362	63082	50mM KCl
N209S	111.69 x 87.37 x 78.92	7359	63079	50mM KCl
R212Q	111.69 x 87.37 x 78.92	7355	62923	50mM KCl

**Supplementary table 7. Oligonucleotides used in this study**
Duplex I; Crystallography
3'-d(CAGGT**Y**CAGAA)-5’ **Y**=OG<br><br>5'-d(TGTCCA**X**GTCT)-3' **X**=1N or THF
Duplex II; Kinetic and Binding experiments
3'-d(GACATTGCCCTCGA**Y**CACCGAGGTACTAGC)-5' **Y**=OG<br><br>5'-d(CTGTAACGGGAGCT**X**GTGGCTCCATGATCG)-3' **X**=A, FA or THF

Supplementary Figures

```
         1       10        20        30        40        50        60
         |       |         |         |         |         |         |
EcMutY   ------------------------------------------------------------
GsMutY   ------------------------------------------------------------
 MUTYH   MTPLVSRLSRLWAIMRKPRAAVGSGHRKQAASQEGRQKHAKNNSQAKPSACDACAGMIAE
 Mutyh   --------------MKKLQASVRS-HKKQPANHKRRRTRALSSSQAKPSSLD--------

EcMutY   ------------------------------MQASQFSAQVLDWYDKYGRKTLPWQI----
GsMutY   -----------------------MTRETERFPAREFQRDLLDWFAR-ERRDLPWRK----
 MUTYH   CPGAPAGLARQPEEVVLQASVSSYHLFRDVAEVTAFRGSLLSWYDQ-EKRDLPWRRRAED
 Mutyh   ------GLAKQKREELLQASVSPYHLFSDVADVTAFRSNLLSWYDQ-EKRDLPWRNLAKE

EcMutY   ----DKTPYKVWLSEVMLQQTQVATVIPYFERFMARFPTVTDLANAPLDEVLHLWTGLGY
GsMutY   ----DRDPYKVWVSEVMLQQTRVETVIPYFEQFIDRFPTLEALADADEDEVLKAWEGLGY
 MUTYH   EMDLDRRAYAVWVSEVMLQQTQVATVINYYTGWMQKWPTLQDLASASLEEVNQLWAGLGY
 Mutyh   EANSDRRAYAVWVSEVMLQQTQVATVIDYYTRWMQKWPKLQDLASASLEEVNQLWSGLGY

                                                                * *
EcMutY   YARARNLHKAAQQVATLHGGKFPETFEEVAA-LPGVGRSTAGAILSLSLGKHFPILDGNV
GsMutY   YSRVRNLHAAVKEVKTRYGGKVPDDPDEFSR-LKGVGPYTVGAVLSLAYGVPEPAVDGNV
 MUTYH   YSRGRRLQEGARKVVEELGGHMPRTAETLQQLLPGVGRYTAGAIASIAFGQATGVVDGNV
 Mutyh   YSRGRRLQEGARKVVEELGGHMPRTAETLQQLLPGVGRYTAGAIASIAFDQVTGVVDGNV

          *                                                 *
EcMutY   KRVLARCYAVSGWPGKKEVENKLWSLSEQVTPAVGVERFNQAMMDLGAMICTRSKPKCSL
GsMutY   MRVLSRLFLVTDDIAKPSTRKRFEQIVREIMAYENPGAFNEALIELGALVCTPRRPSCLL
 MUTYH   ARVLCRVRAIGADPSSTLVSQQLWGLAQQLVDPARPGDFNQAAMELGATVCTPQRPLCSQ
 Mutyh   LRVLCRVRAIGADPTSTLVSHHLWNLAQQLVDPARPGDFNQAAMELGATVCTPQRPLCSH

EcMutY   CPLQNGCIA-----------------------------------AANNSW------ALYP
GsMutY   CPVQAYCQAFAEGVA-----------------------------------------EELP
 MUTYH   CPVESLCRARQRVEQEQLLASGSLSGSPDVEECAPNTGQCHLCLPPSEPWDQTLGVVNFP
 Mutyh   CPVQSLCRAYQRVQRGQL---SALPGRPDIEECALNTRQCQLCLTSSSPWDPSMGVANFP

EcMutY   GKKPKQTLPE-RTGYFLLLQH----EDEVLLAQRPPSGLWGGLYCFPQFADEES------
GsMutY   VKMKKTAVKQVPLAVAVLADD----EGRVLIRKRDSTGLLANLWEFPSCETDGADGKEK-
 MUTYH   RKASRKPPREESSATCVLEQPGAL-GAQILLVQRPNSGLLAGLWEFPSVTWEPSEQLQRK
 Mutyh   RKASRRPPREEYSATCVVEQPGAIGGPLVLLVQRPDSGLLAGLWEFPSVTLEPSEQHQHK

EcMutY   -----LRQWLAQ---RQIAADNLTQ-LTA--FRHTFSHFHLD----------IVPM----
GsMutY   -----LEQMVGE---QYGLQVELTEPIVS--FEHAFSHLVWQLTVFPGRLVHGGPVEE-P
 MUTYH   ALLQELQRWAGP-----LPATHLRH-LGE--VVHTFSHIKLTYQVYGLALEGQTPVTTVP
 Mutyh   ALLQELQRWCGP-----LPAIRLQH-LGE--VIHIFSHIKLTYQVYSLALD-QAPASTAP

EcMutY   ----WLPVSSF-----TGCMDEGNALWYNLAQPPSVG-----LAAPVER-----------
GsMutY   --YRLAPEDELKAYAFPVSHQRVWREYKEWASG---------VRPPP-------------
 MUTYH   PGARWLTQEEFHTAAVSTAMKKVFRVYQGQQPGTCMGSKRSQVSSPCSRKKPRMGQQVLD
 Mutyh   PGARWLTWEEFCNAAVSTAMKKVFRMYEDHRQGTRKGSKRSQVCPPSSRKKPSLGQQVLD

EcMutY   --LLQQLRTGAPV------
GsMutY   -------------------
 MUTYH   NFFRSHISTDAHSLNSAAQ
 Mutyh   TFFQRHIPTDKPNSTTQ—
```

Supplementary Figure 1: Multiple sequence alignment of MutY/MUTYH amino acid sequences. The protein sequences included in the analysis were *Escherichia coli* MutY (EcMutY; NCBI ID ABV07358.1), *Geobacillus stearothermophilus* MutY (GsMutY; WP_013522988.1), human MUTYH (NP_001121897.1) and mouse Mutyh (NP_001153053.1). Catalytic residues are colored in yellow, positions studied herein in green, FSH Loop implied in OG recognition in gray, and IDC region in red. Residues involved in the structural interplay between the [4Fe-4S] cluster and active site are indicated with asterisks. Residues within the Zinc linchpin motif are highlighted in bold letters.

(A) Design of MBP-MUYTH protein for crystallography.

CCATGGGCAAAATCGAAGAAGGTAAACTGGTAATCTGGATTAACGGCGATAAAGGCTATAACGGTCTCGCTGAAGTCGGTAAGAAATTCGA
GAAAGATACCGGAATTAAAGTCACCGTTGAGCATCCGGATAAACTGGAAGAGAAATTCCCACAGGTTGCGGCAACTGGCGATGGCCCTGAC
ATTATCTTCTGGGCACACGACCGCTTTGGTGGCTACGCTCAATCTGGCCTGTTGGCTGAAATCACCCCGGACAAAGCGTTCCAGGACAAGCTG
TATCCGTTTACCTGGGATGCCGTACGTTACAACGGCAAGCTGATTGCTTACCCGATCGCTGTTGAAGCGTTATCGCTGATTTATAACAAAGAT
CTGCTGCCGAACCCGCCAAAAACCTGGGAAGAGATCCCGGCGCTGGATAAAGAACTGAAAGCGAAAGGTAAGAGCGCGCTGATGTTCAACC
TGCAAGAACCGTACTTCACCTGGCCGCTGATTGCTGCTGACGGGGGTTATGCGTTCAAGTATGAAAACGGCAAGTACGACATTAAAGACGTG
GGCGTGGATAACGCTGGCGCGAAAGCGGGTCTGACCTTCCTGGTTGACCTGATTAAAAACAAACACATGAATGCAGACACCGATTACTCCAT
CGCAGAAGCTGCCTTTAATAAAGGCGAAACAGCGATGACCATCAACGGCCCGTGGGCATGGTCCAACATCGACACCAGCAAAGTGAATTAT
GGTGTAACGGTACTGCCGACCTTCAAGGGTCAACCATCCAAACCGTTCGTTGGCGTGCTGAGCGCAGGTATTAACGCCGCCAGTCCGAACAA
AGAGCTGGCAAAAGAGTTCCTCGAAAACTATCTGCTGACTGATGAAGGTCTGGAAGCGGTTAATAAAGACAAACCGCTGGGTGCCGTAGCG
CTGAAGTCTTACGAGGAAGAGTTGGTGAAAGATCCGCGTATTGCCGCCACTATGGAAAACGCCCAGAAAGGTGAAATCATGCCGAACATCC
CGCAGATGTCCGCTTTCTGGTATGCCGTGCGTACTGCGGTGATCAACGCCGCCAGCGGTCGTCAGACTGTCGATGAAGCCCTGAAAGACGCG
CAGACTAATTCGGGATCTGGCAGTGGTTCTGAGAATCTTTATTTTCAGGGCCATATGCAGGCTGCCTCACAGGAAGGCCGCCAGAAACACGC
AAAGAATAACAGTCAGGCAAAACCAAGCGCTTGCGATGGCTTGGCGCGTCAACCGGAGGAAGTCGTGTTACAAGCTAGTGTCTCTTCATATC
ACCTTTTCCGTGACGTTGCAGAAGTTACAGCTTTCCGCGGTTCTTTATTAAGTTGGTATGACCAGGAAAAGCGCGACTTACCTTGGCGTCGCC
GCGCGGAGGATGAAATGGACCTTGACCGTCGTGCTTATGCCGTGTGGGTATCGGAGGTTATGTTGCAACAGACGCAGGTAGCCACCGTGAT
TAACTACTACACGGGTTGGATGCAAAAGTGGCCAACGTTGCAGGATCTGGCGTCCGCTTCACTGGAAGAAGTGAATCAGTTATGGGCAGGA
TTAGGGTACTACTCGCGCGGTCGTCGTCTGCAAGAGGGGGCACGCAAAGTTGTTGAAGAATTGGGAGGCCACATGCCGCGCACTGCAGAAA
CCTTACAACAGTTACTTCCCGGGGTAGGCCGTTATACCGCTGGAGCAATTGCATCCATTGCTTTCGGACAGGCTACTGGAGTAGTTGATGGAA
ATGTGGCACGTGTTTTGTGTCGCGTTCGCGCCATCGGCGCAGACCCTTCATCCACTTTGGTATCTCAGCAGCTGTGGGGATTAGCACAGCAGT
TAGTCGATCCCGCTCGTCCCGGCGACTTCAACCAAGCGGCCATGGAACTTGGTGCCACGGTATGTACGCCACAGCGTCCGCTTTGTAGCCAG
TGTCCGGTTGAGAGCTTGTGTCGTGCTCGCCAACGTGTAGAGCAGGAACAACTTTTAGCAAGCGGTTCCCTGTCTGGCTCTCCCGATGTTGAA
GAATGTGCACCTAACACAGGTCAGTGTCATCTTTGTCTTCCGCCGAGTGAACCCTGGGATCAGACGTTGGGCGTCGTGAACTTTCCGCGTAA
GGCCTCACGCAAACCCCCTCGCGAGGAAAGTAGTGCAACGTGCGTACTGGAGCAACCAGGAGCTTTAGGCGCGCAAATTTTGCTTGTTCAAC
GCCCGAATAGTGGTCTGTTAGCTGGCCTGTGGGAATTTCCATCAGTAACGTGGGAGCCCAGCGAGCAGCTGCAACGCAAAGCCTTATTGCAA
GAGTTACAACGCTGGGCTGGACCGTTGCCAGCGACTCATCTGCGTCATTTAGGAGAAGTAGTACATACGTTCAGTCACATCAAGCTGACCTA
TCAAGTTTATGGCCTTGCCTTAGAAGGGCAAACTCCGGTGACCACCGTGCCGCCCGGCGCACGTTGGCTTACACAAGAAGAATTCCACACGG
CTGCTGTCAGTACGGCGATGAAGAAGGTCTTTCGCGTGTATCAGGGGCAGCAACCTGGAACGTGTATGGGTTCAAAACGTAGTCAAGTATC
GAGTCCATGTTCTCGTAAGAAGCCACGTATGGGTCAGCAGGTCCTGGATAACTTTTTCCGTAGTCATATCTCCACAGACGCCCATAGCTTGAA
CTCGGCGGCCCAGTCAGGCGAGAATCTTTATTTTCAGGGCAGCGGATCAGGGAGCGGATCAGGCAGCGGGCATCACCATCACCATCACCAT
CACTGAGCGGCCGC

(B)

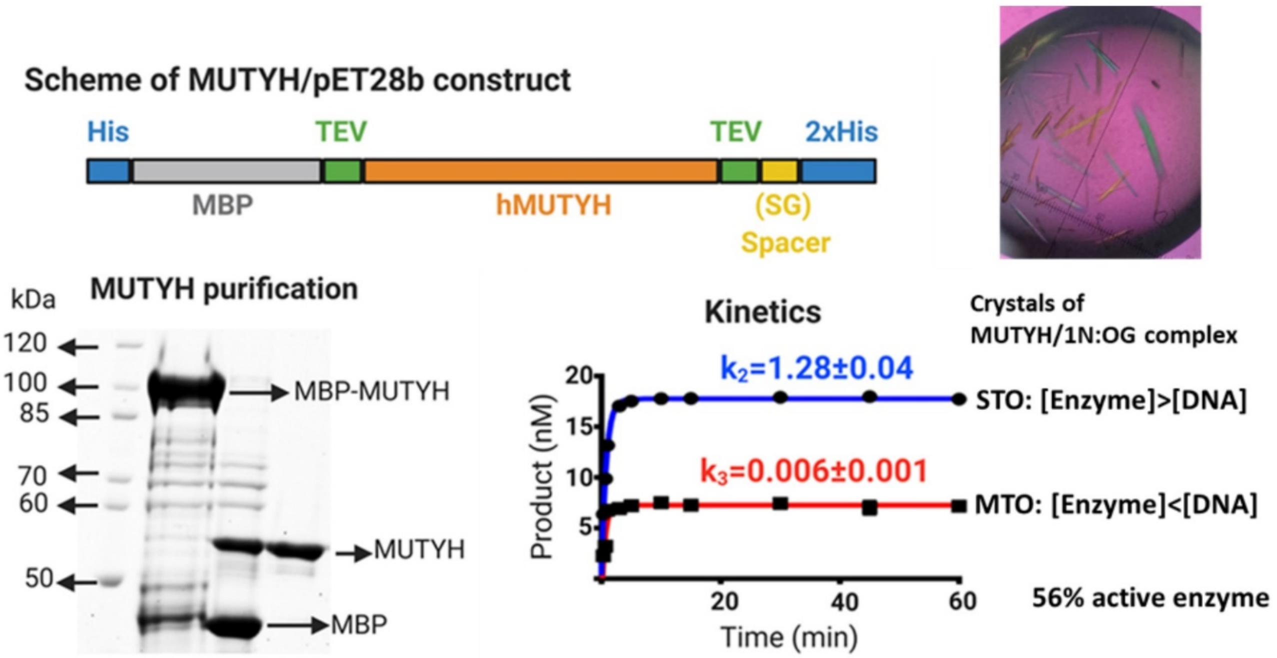

Supplementary Figure 2: Design and details of MUTYH constructs used in this work.

(A) MUTYH construct.

Crystallography. Shown is the open Reading Frame encoding the fusion MBP-MUYTH protein. Highlighted in red and green are the region encoding the Maltose binding protein region and the codon-optimized *MUTYH* gene optimized for its expression in *E. coli*, respectively. A linker with three tandem repeats of Gly-Ser is shown in pink followed by TEV cleavage site in blue. NcoI, NdeI and NotI restrictions sites used for cloning are underlined.

(B) Details of the pET28-MBP-MUTYH construct, MUTYH protein purification and characterization. Upper-left panel; Scheme of the MBP-MUTYH/pET28 construct used in this work. Upper-right panel; Crystals of MUTYH-TSAC. The MUTYH-DNA complex formation for crystallization was carried with 118 µM MUTYH-DNA (1N:OG) complex. The best crystals in terms of size and X-ray diffraction quality were obtain in 0.1 M Bis-Tris [pH 5.5], 0.2 M Ammonium Sulphate and 20% PEG 3350. Lower-left panel; results of the purification of MUTYH protein. First lane, molecular weight ladder. Second lane, elution fraction after Nickel affinity chromatography. Third lane, Elution fraction after TEV cleavage step. Fourth lane, pure MUTYH after Heparin affinity chromatography. Lower-right panel. Biochemical characterization of MUTYH without tags or fusion MBP protein.

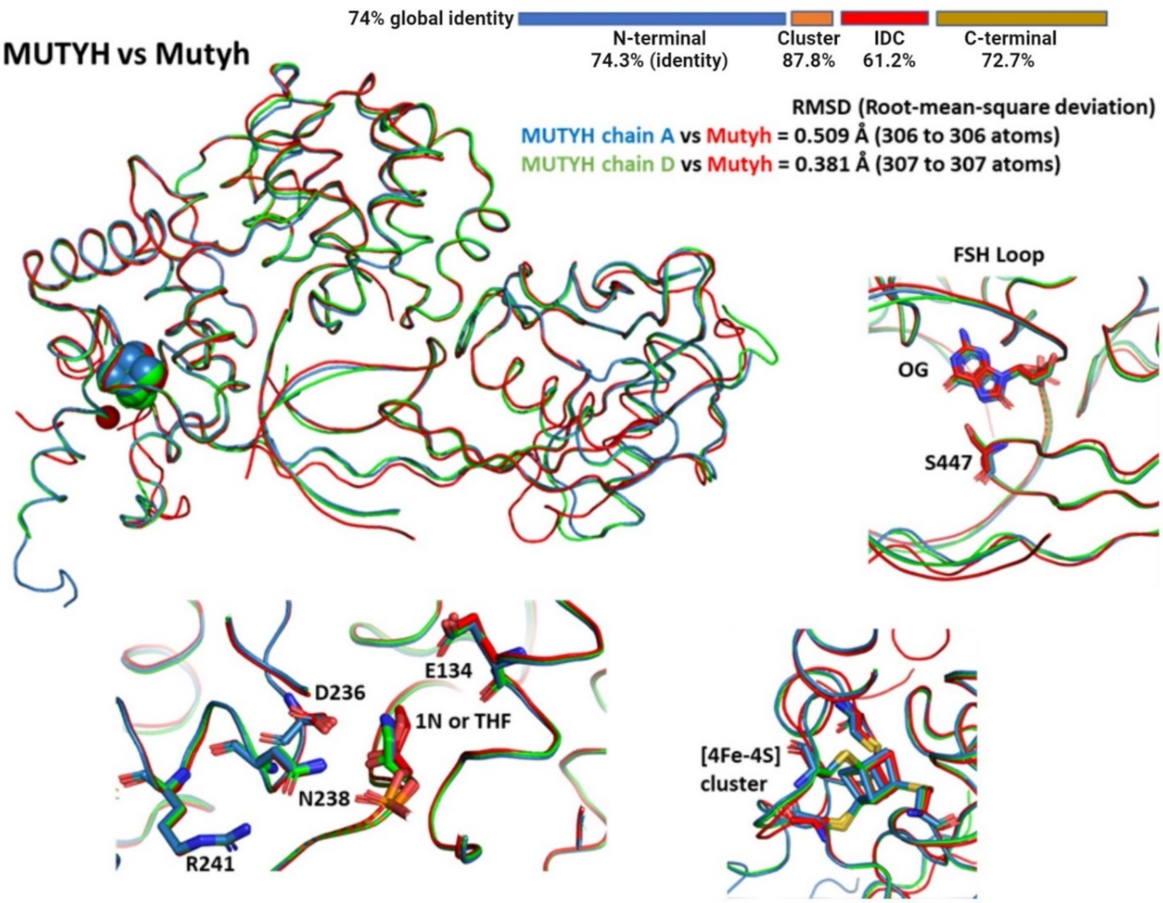

Supplementary Figure 3: Structural comparison of Mutyh/MUTYH. Structural alignment including both copies of MUTYH-DNA complexes included the unit cell and mouse Mutyh (PDB ID; 7EF8)[1]. A close-up view of the catalytic residues, OG recognition sphere and [4Fe-4S] cluster is shown. Moreover, a schematic amino acid sequence analysis is displayed indicating percent identity per regions. Both structures, in spite of being only 74% identical in amino acid sequence, aligned with a RMSD of up to 0.381 Å. However, in the IDC region the identity drops to 61% (Figure S1) and this may impact the structure, flexibility and Zn lability that results in the difference localized here between the MUTYH and mouse Mutyh structures.

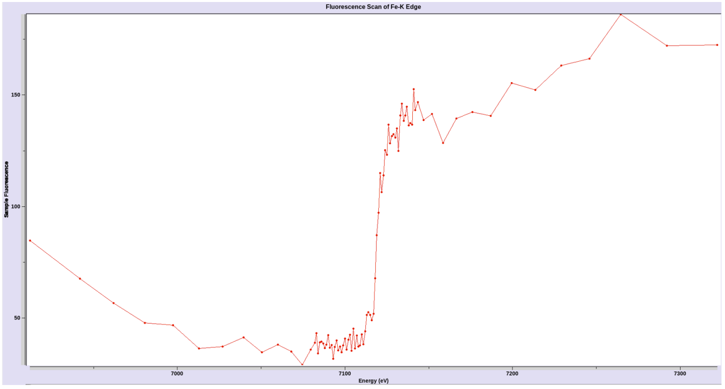

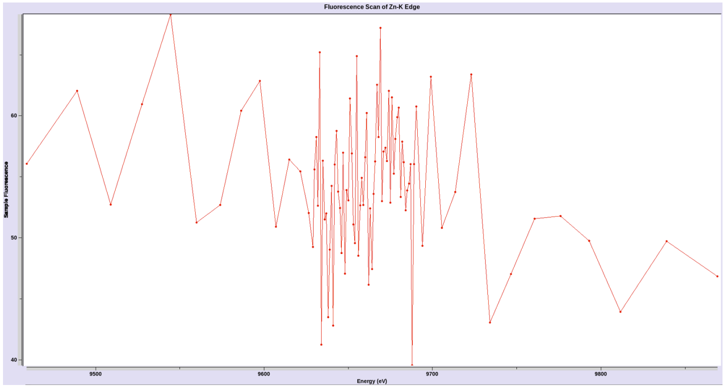

Supplementary Figure 4. X-ray absorption near-edge spectra of the iron of the [4Fe–4S] cluster (upper panel) and the Zn (lower panel) of MUTYH crystal.

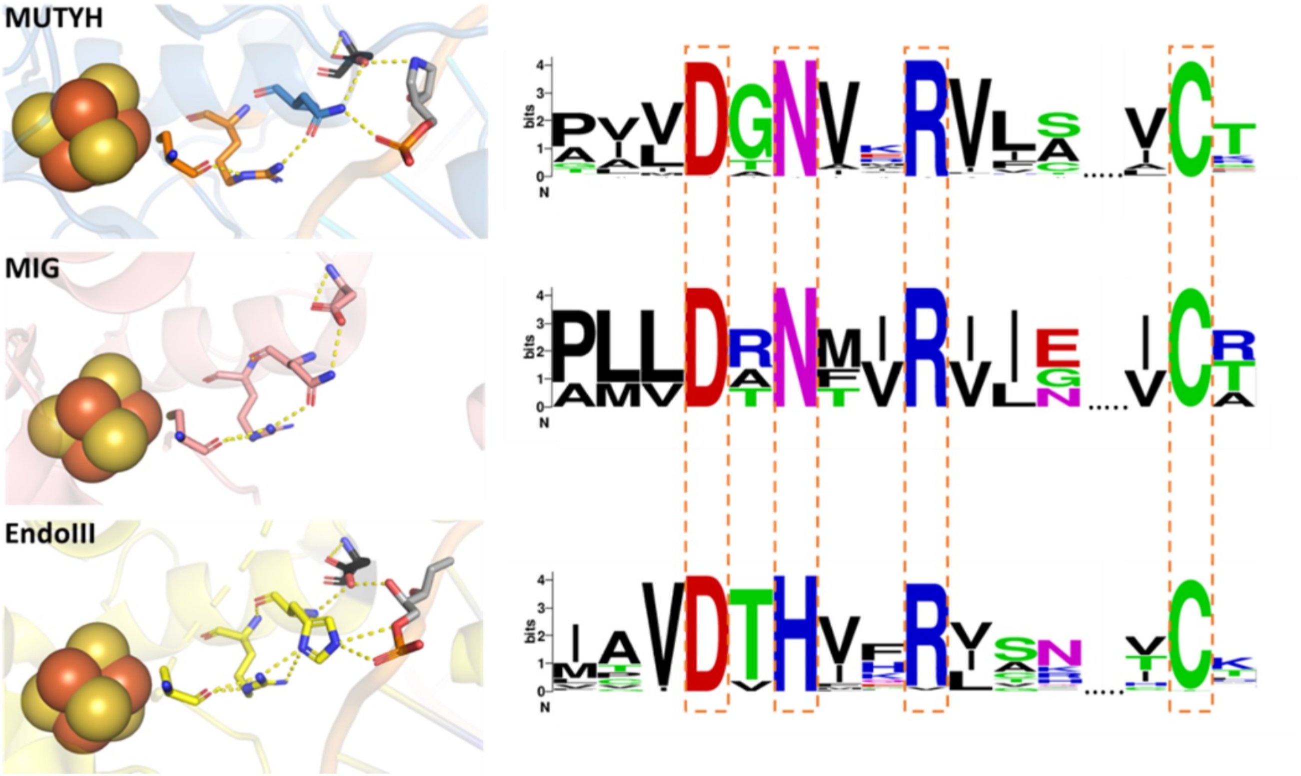

Supplementary Figure 5.- Conservation of the residues that participate in the allosteric network connecting the [4Fe-4S] cluster and the active site in Helix-hairpin-Helix DNA glycosylases. A close-up view of the residues involved in the structural connectivity is shown. The H-bond network is shown in yellow dotted lines. The structures shown are human MUTYH, MIG (PDB ID 1KEA)[7] and EndoIII (1ORP)[8]. On the right side a logo sequence of the corresponding region involved in the interplay highlighting the residues that participates in the H-bond network (orange boxes).

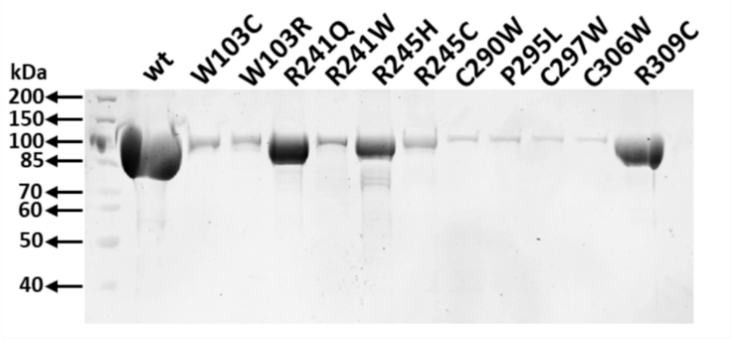

Supplementary Figure 6. SDS-PAGE showing the MBP-MUTYH variants used in this work.

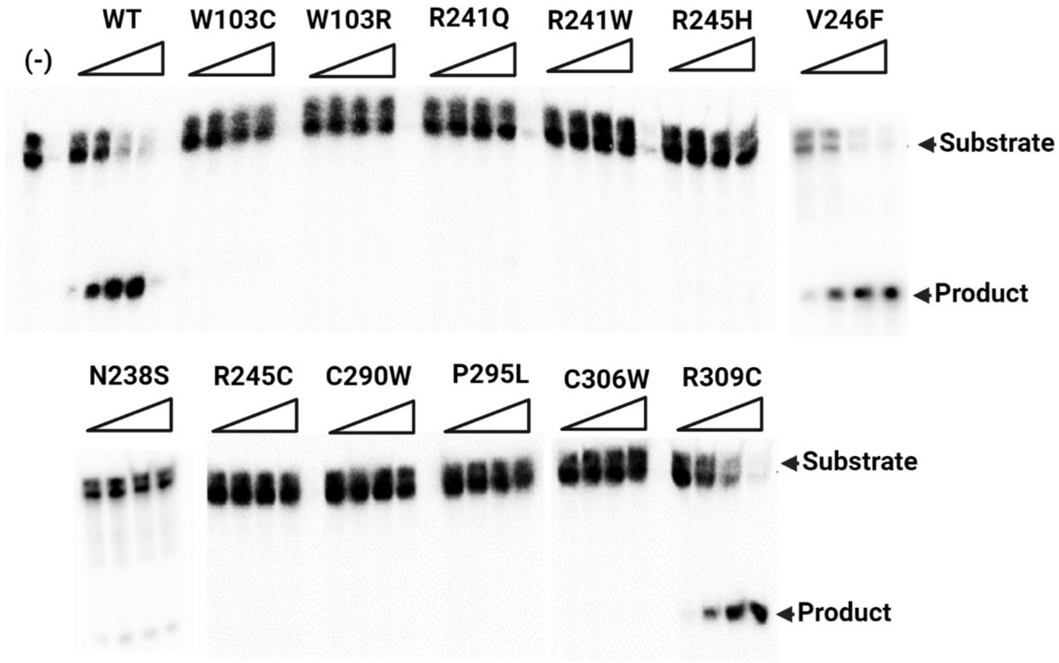

Supplementary Figure 7. Qualitative glycosylase characterization of MBP-MUTYH cancer-associated mutants. Condition used: 20 nM of A:OG-containing DNA duplex was titrated with increasing concentrations of MBP-MUTYH (10-250 nM) for 1 h at 37 °C. The reactions were resolved by UREA-PAGE and visualized by storage phosphor autoradiography.

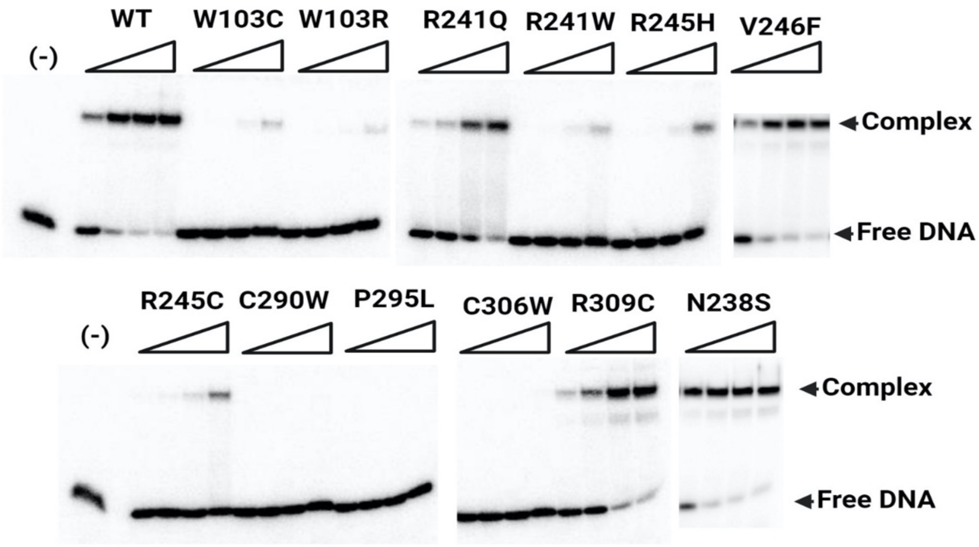

Supplementary Figure 8. Qualitative binding assays. 20 nM of THF:OG-containing DNA duplex was titrated with increasing concentrations of MBP-MUTYH (6.25-400 nM) for 20 min at 25 °C. Binding reactions were resolved by native-PAGE

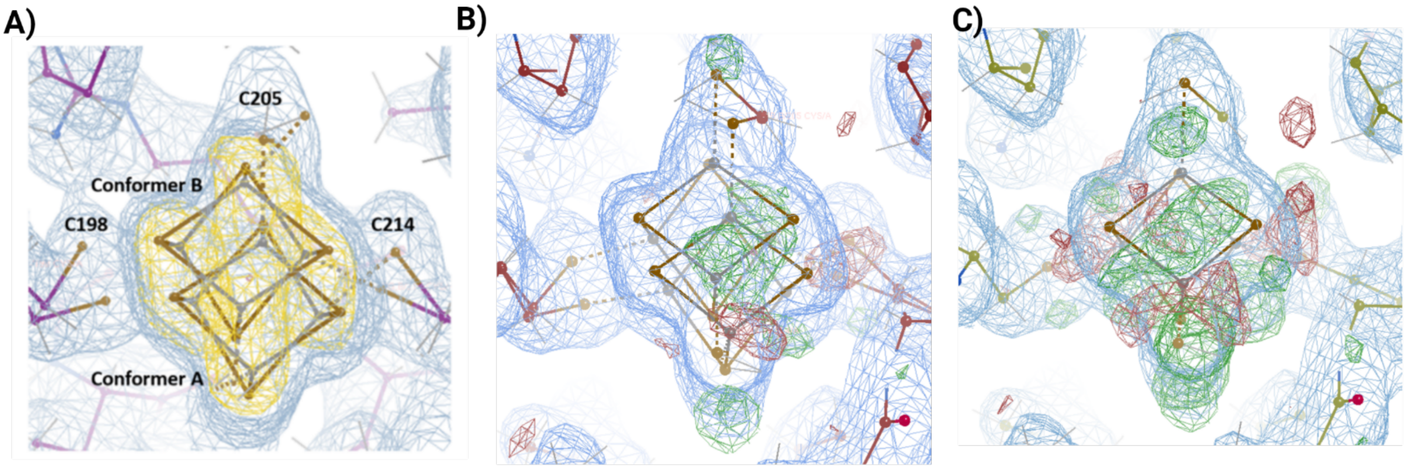

Supplementary Figure 9.- Structural analysis of the alternative conformations of the [4Fe-4S] cluster within the R149Q GsMutY-THF:OG structures. The 2|Fo|–|Fc| omit map (blue) was calculated to the 1.58 Å resolution limits, and contoured at 1.0 rmsd. A) The ANOM map contoured to 5.0 rmsd (gold) shows elongated anomalous signal for the [4Fe-4S] cluster indicating alternative conformations of the cofactor. Difference map of B) [4Fe-4S] cluster motif modeling two conformation of the cofactor and C) after removing the A conformer.

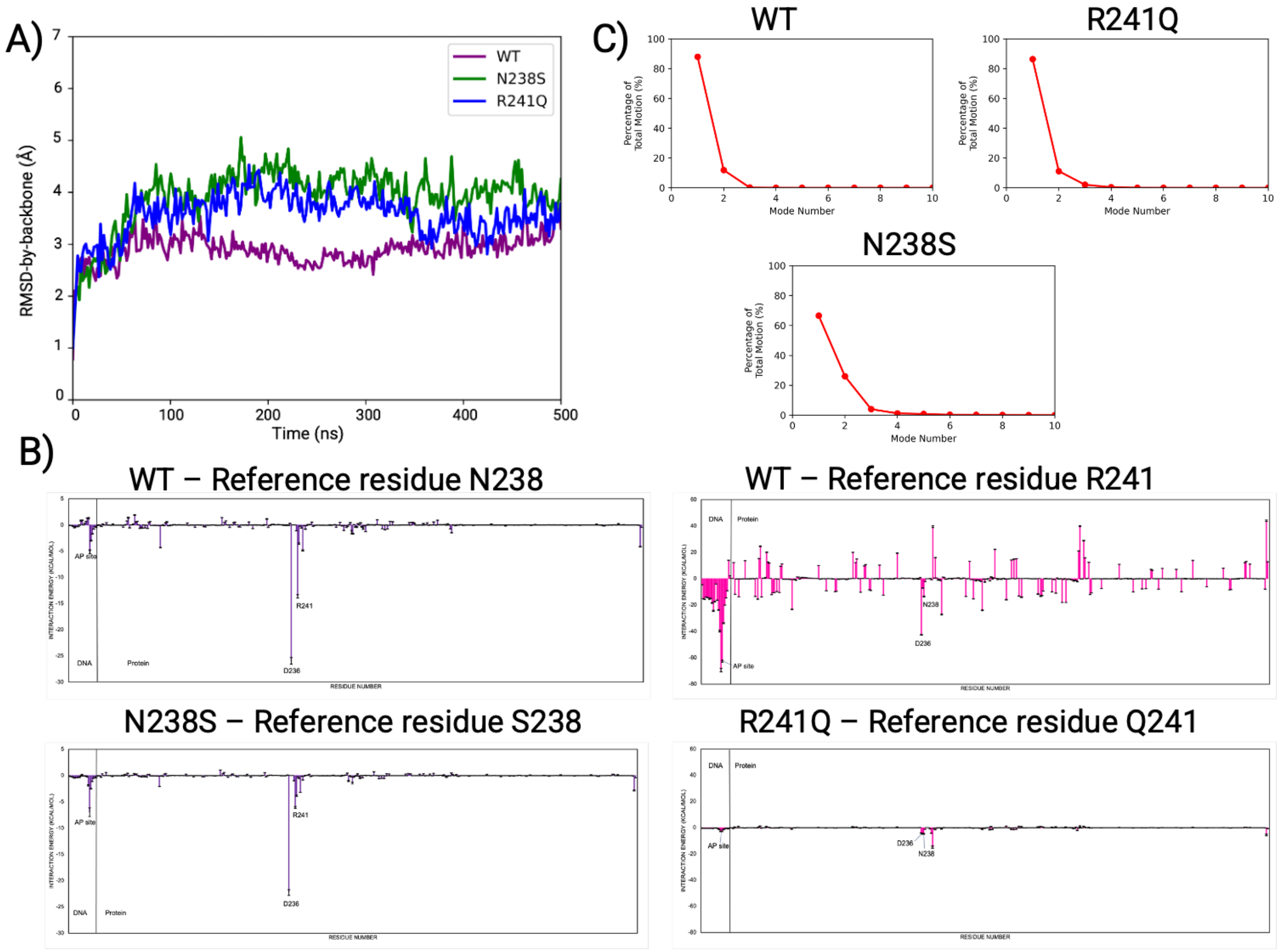

Supplementary Figure 10: Molecular dynamic simulations of WT MUTYH, N238S and R241Q cancer-associated variants in human structure. A) Root Mean Square Deviation (RMSD), B) Energy Decomposition Analysis (EDA) with respect to residue Asn238 (in purple), with respect to residue Arg241 (in pink), C) Normal Mode Analysis (NMA) – Percentage contribution to total motion by each mode number

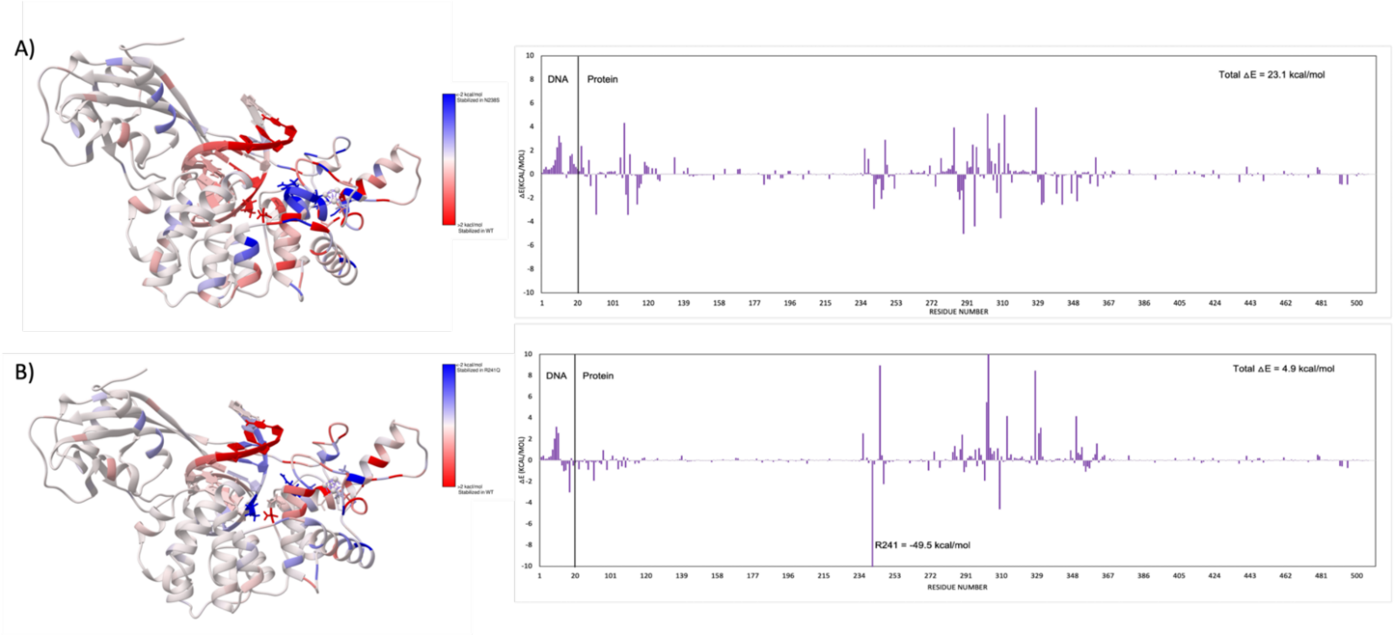

Supplementary Figure 11. - Energy decomposition analysis of the nonbonded interactions of the MUTYH human structure with respect to the [4Fe-4S] cluster. The energy difference between (A) N238S and WT and (B) R241Q and WT are shown. Residues highlighted in red color and blue color represent the interaction energies ≥ +2 or ≤ -2 kcal mol^{-1}.

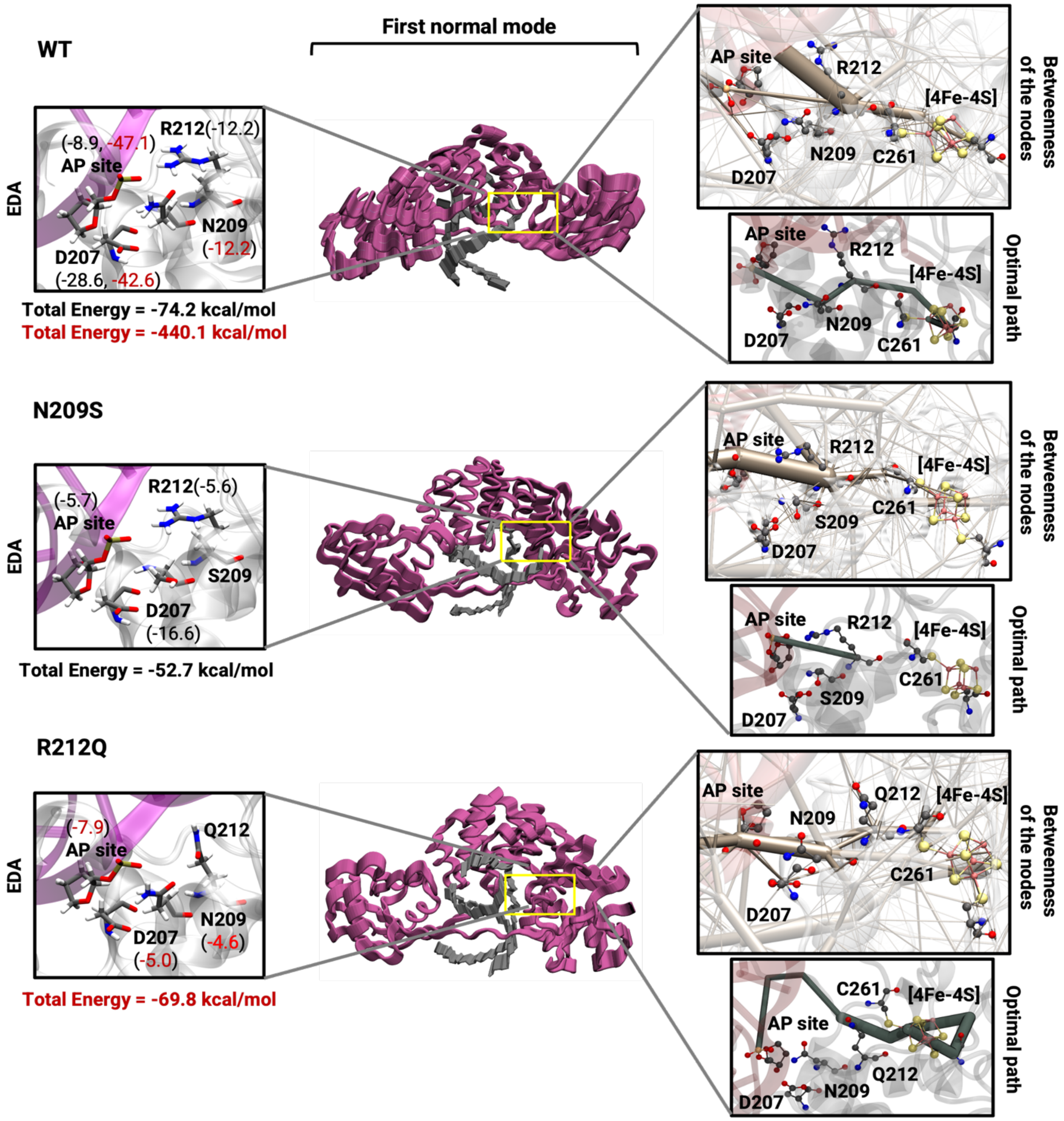

Supplementary Figure 12.- Molecular dynamic simulations of WT, N209S and R212Q variants in mouse Mutyh structure. The Energy Decomposition Analysis (EDA) (Left panel) shows the intermolecular non-bonded interactions (Coulomb and Vander Waals interactions; kcal/mol) between Asn209 or Arg212 (black and red values, respectively) and the rest of the residues involved. For the Network Analysis the betweenness of the nodes involved in the multi-motif bridge is shown in brown color (Right-upper panel) and the optimal path between the AP site and [4Fe-4S] cluster is displayed in green (Right-lower panel). The first mode analysis is shown on the middle where the Mutyh and DNA are illustrated in magenta and gray, respectively. Slightly different networks are observed in mouse Mutyh structure compared to human MUTYH structure. The right panel shows the betweenness of nodes in brown (upper panel) and the optimal path between AP site and Fe-S cluster in dark green (lower panel). In the WT structure, the AP site exhibits contacts with D207, N209 and somewhat stronger contact with R212. Conversely, the N209S mutant system shows a high betweenness, establishing a strong contact between AP site and R212. Furthermore, a strong connection is observed between AP site and N209 in the R212Q mutant structure while a connection is maintained between AP site and catalytic residue D207. Additionally, the optimal path between AP site and Fe-S cluster in the WT structure involves N209 and R212. This path is disrupted in N209S

mutant structure and alternative path through the protein takes place without involving the residues in the H-bond bridge in R212Q mutant structure.

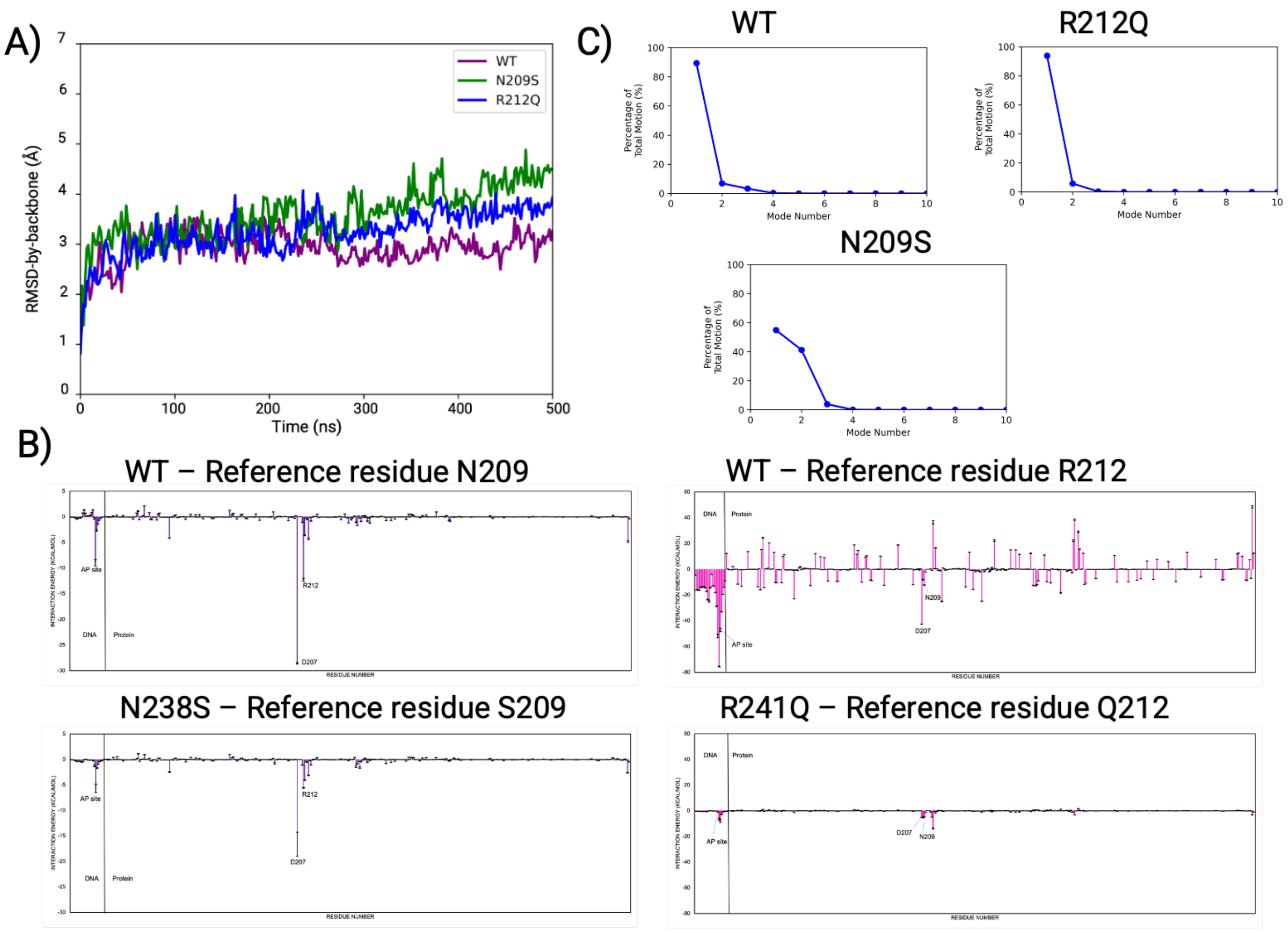

Supplementary Figure S13.- Additional results of the molecular dynamic simulations of WT, N209S and R212Q cancer-associated variant in mouse Mutyh structure. A) Root Mean Square Deviation (RMSD), B) Energy Decomposition Analysis (EDA) with respect to residue Asn209 (in purple), with respect to residue Arg212 (in pink), C) Normal Mode Analysis (NMA) – Percentage contribution to total motion by each mode number.

Supplementary Methods:

Synthesis of 1N nucleoside

Synthesis of 1N (**7**) involves a six-step synthesis with dibenzylamine (**1**) as the starting material (**Scheme 1).** Modifications of the published synthesis[10] were improved workup of **2**, a new method for reducing **6** to **7** via the H-Cube Mini Plus from ThalesNano, and a new approach to the purification of **7**. The first step of the 1N synthesis involves a Noyori Oxidation of dibenzylamine to form a nitrone intermediate that is hydrolyzed under acidic conditions to form a *N*-benzylhydroxylamine•HCl salt (**2**) (**Scheme 1**). To more effectively remove a benzaledyde side product, a simple-distillation setup was used to obtain a more effective removal of benzaldehyde through steam distillation. With the installation of an ice-cold base trap, the setup also addressed the concerns of expelling HCl. A series of steam distillations were performed at atmospheric pressure. Benzaldehyde, with a boiling point of 178.1°C, was successfully removed concurrently with water at 80°C. Toluene was then added to accelerate the evaporation of water. Following,

toluene was removed through rotary evaporation and the crude product was subject to washing with toluene. With this protocol, the recrystallized product obtained was white in color, an indication that the benzaldehyde was effectively removed.

The final step of the synthesis requires the hydrogenation of 1N-Bn (**6**) to yield 1N (**7**). Previously this was achieved by refluxing solution of **6** to H_2(g) with 10% palladium on carbon (Pd/C) as catalyst. However, use of a hydrogenator, the H-Cube Mini Plus from ThalesNano, was used as an alternative to convert **6** into **7**. Using the H-cube brings the advantage of efficiency to the synthesis. The *N*-benzyl group of **6** was hydrogenated at 80°C, with a 1 bar supply of H_2(g), using a 10% Pd/C catalyst cartridge. With a flow rate of 1 mL/min, a 6 mL sample of 20 mM 1N-Bn was fully converted to 1N in about 15 minutes, including setup and cleanup of the equipment. Lastly, preparative TLC was used for the final purification of 1N. Briefly, 1N was spotted on a 12.5×25 cm TLC plate. The plate was placed in a chamber with neat MeOH. Samples of the first two spots were collected by scraping off the silica and extracting the product(s) by stirring in neat MeOH for 12 h. ESI-MS confirmed that the second spot from the starting line contained 1N (R_f=0.17).

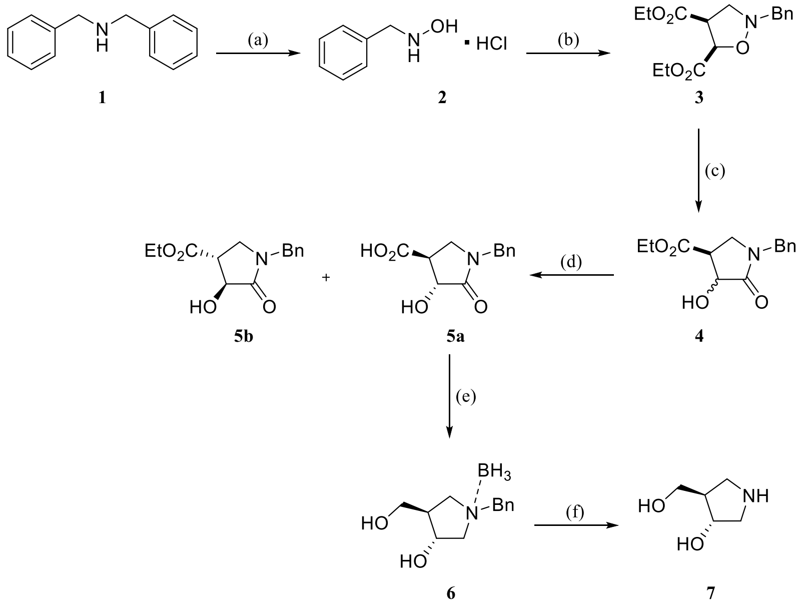

Scheme 1 1N synthetic scheme. (a) 1. $Na_2WO_4 \bullet 2H2_2O$, H_2O_2, MeOH, rt 18h, 2. HCl (aq.) (b) diethylmaleate, CH_2O, EtOH, reflux 2.5 h (c) Zn, AcOH, rt 8 h (d) Cal-B lipase, phosphate buffer (0.5 M pH 7.5), 30°C 24 h (e) $BH_3 \bullet$DMS, THF, reflux 11 h (f) 10% Pd/C, H_2, MeOH (anh.), 60°C 12h (2x).

SUPPLEMENTARY REFERENCES

1. Nakamura, T. et al. Structure of the mammalian adenine DNA glycosylase MUTYH: insights into the base excision repair pathway and cancer. *Nucleic acids research* **49**, 7154-7163 (2021).
2. Fokkema, I.F. et al. The LOVD3 platform: efficient genome-wide sharing of genetic variants. *European Journal of Human Genetics* **29**, 1796-1803 (2021).
3. Forbes, S.A. et al. COSMIC: somatic cancer genetics at high-resolution. *Nucleic acids research* **45**, D777-D783 (2017).
4. Demir, M. et al. Structural snapshots of base excision by the cancer-associated variant MutY N146S reveal a retaining mechanism. *Nucleic Acids Research* (2023).
5. Russelburg, L.P. et al. Structural Basis for Finding OG Lesions and Avoiding Undamaged G by the DNA Glycosylase MutY. *ACS chemical biology* **15**, 93-102 (2019).
6. Woods, R.D. et al. Structure and stereochemistry of the base excision repair glycosylase MutY reveal a mechanism similar to retaining glycosidases. *Nucleic acids research* **44**, 801-810 (2016).
7. Mol, C.D., Arvai, A.S., Begley, T.J., Cunningham, R.P. & Tainer, J.A. Structure and activity of a thermostable thymine-DNA glycosylase: evidence for base twisting to remove mismatched normal DNA bases. *Journal of molecular biology* **315**, 373-384 (2002).

8. Fromme, J.C., Banerjee, A., Huang, S.J. & Verdine, G.L. Structural basis for removal of adenine mispaired with 8-oxoguanine by MutY adenine DNA glycosylase. *Nature* **427**, 652-656 (2004).
9. Diederichs, K. & Karplus, P.A. Improved R-factors for diffraction data analysis in macromolecular crystallography. *Nature structural biology* **4**, 269-275 (1997).
10. Chu, A.M., Fettinger, J.C. & David, S.S. Profiling base excision repair glycosylases with synthesized transition state analogs. *Bioorganic & medicinal chemistry letters* **21**, 4969-4972 (2011).
