# Supplementary material for: Structure of human MUTYH and functional profiling of cancer-associated variants reveal an allosteric network between its [4Fe-4S] cluster cofactor and active site required for DNA repair
Source: Nat Commun. 2025 Apr 16;16:3596. doi: 10.1038/s41467-025-58361-w (PMC12000561; doi:10.1038/s41467-025-58361-w)
Supplement: Supplementary file 4 — Description of Supplementary Data files [file 41467_2025_58361_MOESM4_ESM.docx]

**Description of additional Supplementary data files**

File Name: Supplementary Data 1

Description: A multiple sequence alignment including 687 amino acid sequence of Archaea, Bacteria and Eukaryote MutY homologs was generated using the MUSCLE algorithm as implemented with the Geneious software package (Version 4.8, Biomatters).
